# Humoral Immune Responses of Dengue Fever Patients Using Epitope-Specific Serotype-2 Virus-Like Particle Antigens

**DOI:** 10.1371/journal.pone.0004991

**Published:** 2009-04-01

**Authors:** Wayne D. Crill, Holly R. Hughes, Mark J. Delorey, Gwong-Jen J. Chang

**Affiliations:** Arbovirus Diseases Branch, Division of Vector-Borne Infectious Diseases, Centers for Disease Control and Prevention, Public Health Service, U. S. Department of Health and Human Service, Fort Collins, Colorado, United States of America; Institute of Molecular and Cell Biology, Singapore

## Abstract

Dengue virus (DENV) is a serious mosquito-borne pathogen causing significant global disease burden, either as classic dengue fever (DF) or in its most severe manifestation dengue hemorrhagic fever (DHF). Nearly half of the world's population is at risk of dengue disease and there are estimated to be millions of infections annually; a situation which will continue to worsen with increasing expansion of the mosquito vectors and epidemic DF/DHF. Currently there are no available licensed vaccines or antivirals for dengue, although significant effort has been directed toward the development of safe and efficacious dengue vaccines for over 30 years. Promising vaccine candidates are in development and testing phases, but a better understanding of immune responses to DENV infection and vaccination is needed. Humoral immune responses to DENV infection are complex and may exacerbate pathogenicity, yet are essential for immune protection. In this report, we develop DENV-2 envelope (E) protein epitope-specific antigens and measure immunoglobulin responses to three distinct epitopes in DENV-2 infected human serum samples. Immunoglobulin responses to DENV-2 infection exhibited significant levels of individual variation. Antibody populations targeting broadly cross-reactive epitopes centered on the fusion peptide in structural domain II were large, highly variable, and greater in primary than in secondary DENV-2 infected sera. E protein domain III cross-reactive immunoglobulin populations were similarly variable and much larger in IgM than in IgG. DENV-2 specific domain III IgG formed a very small proportion of the antibody response yet was significantly correlated with DENV-2 neutralization, suggesting that the highly protective IgG recognizing this epitope in murine studies plays a role in humans as well. This report begins to tease apart complex humoral immune responses to DENV infection and is thus important for improving our understanding of dengue disease and immunological correlates of protection, relevant to DENV vaccine development and testing.

## Introduction

Dengue virus (DENV) is the quintessential 21^st^ century re-emerging infectious disease. Advances in post exposure treatment, epidemiological understanding, and vector control did much to reduce dengue disease burden in the past. However, in the last three decades DENV has spread epidemically; dramatically increasing in disease severity and range with overlapping co-circulation of the four DENV serotypes spreading into geographic regions containing other pathogenic flaviviruses [1], [2], [3]. Approximately 40% of the world's population, over 2.5 billion people, live at risk of infection in DENV-endemic areas resulting in estimated millions of infections annually [4], [5]. Significant effort and resources have been applied toward DENV vaccine development over the last 30 years, yet in spite of promising vaccine candidates in development and/or early-phase trials, a safe and efficacious vaccine appears to still be years away [5], [6].

**Figure 1:**
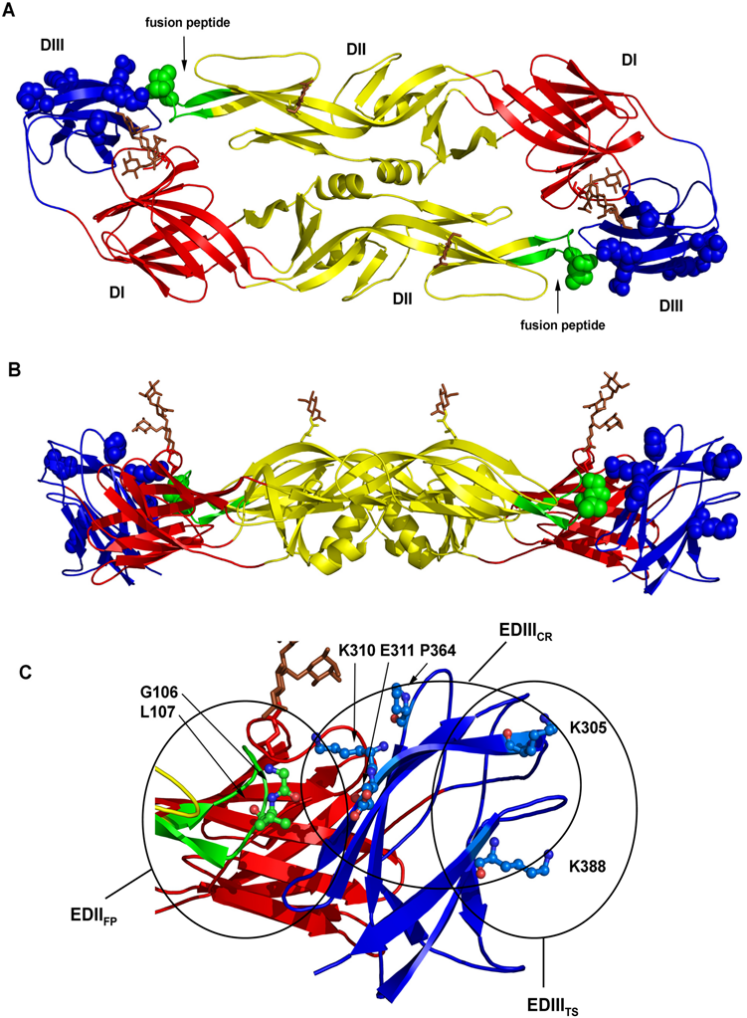
Structural locations of envelope (E) protein epitope-specific knock-out substitutions. (A) Crystal structure of the DENV-2 E protein dimer [15] as it appears from above in mature virions and depicted as a ribbon diagram. The structural domains are colored red (EDI), yellow (EDII), and blue (EDIII). The highly conserved fusion peptide, located at the distal end of EDII is colored green and the glycans in EDI (N153) and EDII (N67) are depicted as ball and stick representation and colored brown. Epitope specific knock-out substitutions in the fusion peptide and in EDIII are depicted as space filling representations. (B) Side view of the same representation of the E protein dimer in mature virions with all structural depictions and colors the same as in panel A. (C) An enlarged view from panel B of the interface between the EDII fusion peptide of one E monomer and EDIII of the alternate monomer of the E dimer. EDII fusion peptide (EDII_FP_), EDIII cross-reactive (EDIII_CR_), and EDIII DENV-2 serotype specific (EDIII_TS_) antigenic regions are noted and encircled. Residues locations of epitope-specific knock out substitutions utilized in this study are depicted as ball and stick representations. Substitutions of Gly106 and Leu107 in the EDII fusion peptide knock out the binding of broadly cross-reactive immunoglobulins, those recognizing viruses in the DENV complex and other flavivirus complexes. Substitutions of Lys310, Glu311, Pro364, and Lys388 in EDIII knock out the binding of immunoglobulins recognizing all or subsets of the four serotypes of DENV, but do not interfere with DENV-2 specific immunoglobulin recognition. Substitutions of Lys305 knock out the binding of DENV-2 serotype specific MAbs.

DENV consist of four closely related viral serotypes (DENV-1, -2, -3, and -4) and as with the other flaviviruses, infection with any single virus appears to provide life-long immunity with cross-protection to other DENV serotypes being limited and transient [7], [8], [9], [10]. Human infections with DENV range from asymptomatic to an acute self-limiting febrile illness known as dengue fever (DF) or with increasing frequency, a life-threatening hemorrhagic fever and circulatory shock known as dengue hemorrhagic fever/dengue shock syndrome (DHF/DSS) [2]. The DENV genome is a positive-sense single-stranded RNA molecule, approximately 11 kb in length. It is transcribed as a single polyprotein encoding three structural proteins; capsid (C), premembrane/membrane (prM/M) and envelope (E) proteins and seven non-structural proteins [11]. Mature virions contain an ER derived lipid bilayer covered with a dense lattice of membrane-bound prM/M and E proteins, organized into dimmers on its surface [12]. The E protein is the primary protective antigen containing a highly conserved internal fusion peptide and the cellular receptor-binding motifs, both essential for viral infectivity via receptor-mediated endocytosis [11], [12], [13], [14]. DENV and all other flavivirus E proteins contain three structural and functional domains [15], [16], [17]. The epitope specificity and biological characteristics of antibody responses to the E protein are almost entirely deduced from murine MAb studies. E protein domain I (ED1) is the central domain and contains both virus-specific and cross-reactive, predominately non-neutralizing epitopes; EDII, the dimerization domain, contains the internal fusion peptide which forms the epicenter of a series of overlapping immunodominant cross-reactive epitopes stimulating predominately non- or weakly neutralizing antibodies; EDIII has an immunoglobulin-like fold, contains the primary cellular receptor-binding motifs and in mice elicits virus-specific, highly protective neutralizing antibodies and DENV complex cross-reactive antibodies [18], [19], [20], [21], [22], [23], [24], [25] (Fig. 1).

Infection with any DENV serotype thus produces a wide spectrum of anti-E immunoglobulins varying from broadly cross-reactive - recognizing all flaviviruses, to those recognizing only subsets of viruses in different serocomplexes, those recognizing only DENV-complex viruses to DENV serotype-specific antibodies. The broadly cross-reactive antibodies stimulated from the overlapping immunodominant epitopes surrounding the EDII fusion peptide are predominantly non-neutralizing as evidenced by the general lack of cross-protection between the flaviviruses [7], [8], [9], [10], [19], [26]. DENV complex and sub-complex cross-reactive antibodies stimulated from overlapping epitopes in EDIII vary in their neutralizing capabilities [18], [22], [24] and these antibodies can be protective as evidenced by occasionally documented transient cross-protection between DENV serotypes [9]. Secondary infection with a heterologous DENV serotype stimulates a broadly cross-reactive anamnestic immune response, which although not efficiently cross-protective, can cause serious public health issues. The presence of cross-reactive anti-E immunoglobulins in recovered human sera can create difficulty for serodiagnosis and especially for estimates of disease burden in regions with multiple co-circulating pathogenic flaviviruses. In clinical serodiagnosis detecting IgM, this cross-reactivity is most problematic with secondary infections due to the difficulty of differentiating antibodies elicited by primary infections from those elicited in a secondary infection [27], [28], [29]. For example, in Puerto Rico since 1997 all four DENV serotypes were co-circulating and the vast majority of patient sera were secondary DENV infections. With the arrival of West-Nile virus (WNV) into the Caribbean and apparent DENV infections with neurological manifestations, clinical serodiagnosis has become especially complicated (CDC Dengue Branch pers. com., [30], [31]). Serosurvey estimates of flavivirus disease burden are even more problematic due to cross-reactive IgG responses accumulated over a lifetime of multiple virus or vaccination exposures.

The presence of cross-reactive anti-E immunoglobulins may enhance the pathogenicity of flavivirus infections via the process of antibody-dependent enhancement (ADE) of infection [32]. Although ADE has been demonstrated in vitro for encephalitic as well as hemorrhagic flaviviruses [33] it is of greatest in vivo concern with DENV infections where the severe pathogenic manifestations DHF/DSS are correlated with secondary infections and sub-neutralizing levels of heterologous anti-E immunoglobulins [34], [35], [36]. Concern over ADE and its role in DHF/DSS has instilled a belief in the necessity for tetravalent DENV vaccines stimulating balanced immune responses across DENV serotypes to reduce this concern [6], [37], [38]. However, multivalent DENV candidate vaccine trials have been severely hampered by difficulties in inducing a balanced serotype-specific immune responses in vaccinees [37].

An important research agenda necessary to successfully address the DENV global public health challenge is to improve our understanding of humoral immune responses to DENV infection; specifically the E-protein epitope-targeting of the immunoglobulin response and the relative quantities of epitope-specific antibody populations after viral exposure and vaccination in humans, the infection neutralizing and/or enhancing capabilities of these immunoglobulin populations and their role as correlates of protection in vaccine efficacy studies [4], [22], [34], [39], [40], [41], [42]. As an initial step toward this goal, in this report we develop a series of E-protein epitope-specific knock-out virus-like particles (VLPs) and use these antigens to measure humoral immune responses in DENV-2 infected human sera from Puerto Rico and Taiwan. We quantify immunoglobulin populations targeting four distinct E-protein antigenic regions: the most broadly cross-reactive EDII fusion peptide epitopes, EDIII serocomplex cross-reactive epitopes, epitopes incorporating determinates from either or both of these domain regions, and EDIII DENV-2 type-specific epitopes. Humoral immune responses in DENV-2 infected patient sera were highly variable between individual sera and immunoglobulin class. Immunoglobulin populations recognizing dominant cross reactive epitopes centered on the EDII fusion peptide were large and highly variable, averaging approximately 50% and 30% in primary and secondary DENV-2 infected sera respectively. Immunoglobulin recognizing DENV complex cross-reactive epitopes in EDIII exhibited similar trends but the magnitudes were smaller overall and the EDIII cross-reactive IgM response was much greater than IgG in both primary and secondary DENV-2 infected sera. Immunoglobulin populations recognizing EDIII DENV-2 specific epitopes were very small though again larger in IgM than in IgG. IgG recognizing this epitope averaged only 1% of the immunoglobulin response yet the magnitudes of EDIII virus-specific IgG were significantly correlated with DENV-2 specific neutralization regardless of primary or secondary infection status. The results presented in this report extend our understanding of the diverse nature of humoral immune responses to human DENV infection and we discuss their relevance in the context of understanding DENV disease and the development of safe and efficacious DENV vaccine candidates.

## Results

### Monoclonal Antibody Epitope Mapping

We examined the effects of substitutions in two distinct antigenic regions of the E-glycoprotein, EDII and EDIII on MAb reactivities (Fig. 1). The highly conserved fusion peptide located in EDII is an immunodominant region containing multiple overlapping epitopes typically eliciting weakly neutralizing and widely cross-reactive antibodies against viruses from several major serocomplexes [19], [20], [43], [44], [45]. DENV EDIII elicits predominately complex and sub-complex cross-reactive and virus-specific antibodies, some of which can be potently neutralizing [18], [22], [24]. Previous studies of flavivirus epitope mapping have demonstrated that different substitutions of an E-protein residue can have distinct effects on both MAb reactivities and VLP secretion, and that a specific substitution's effect on MAb binding can vary between flaviviruses [14], [19], [43], [44], [46]. For this reason we chose first to determine the effect of individual E-protein substitutions on MAb reactivity and VLP secretion, and then to combine substitutions in an attempt to maximize both MAb reactivity reductions and VLP secretion.

In the fusion peptide of EDII we examined two, five and six different substitutions at Gly104, Gly106 and Leu107 respectively (Fig. 1 and Table 1). As previously observed with different substitutions and with other viruses, G104 substitutions dramatically reduced VLP secretion from transiently transformed COS-1 cells (Table 1, [20], [43], [44]). Individual substitutions for G106 or L107 residues reduced the reactivities predominately against group-cross-reactive MAbs which recognize all flavivirus E proteins (Tables 1 and 2, some data not shown). Based upon these observations we combined three different substitutions at G106 with two at L107 to create six double-mutant constructs. As expected, the observed effects of the single substitutions on MAb reactivities and VLP secretion typically combined additively into the double mutants. In addition to altering the most broadly cross-reactive MAbs, we also observed reactivity reductions for DENV complex and subcomplex reactive MAbs D3-5C9-1, 9D12, 10A4D-2, and 1B4C-2 (Tables 1 and 2, some data not shown). In general, reactivity reductions to these MAbs were less pronounced than those observed with the more broadly cross-reactive MAbs. Thus, combinations of substitutions acting as epitope determinates in the EDII fusion peptide have a greater effect than do individual substitutions on disrupting the binding of some antibodies in this region. In DENV-2, fusion peptide residues act as epitope determinates for antibodies with diverse patterns of cross reactivity; predominately flavivirus group cross-reactive antibodies, but also for MAbs recognizing only individual viruses from different flavivirus serocomplexes, to DENV complex cross-reactive MAbs. This appears to be a general phenomenon, as recent reports in other flaviviruses demonstrate that antibodies exhibiting varying levels of cross-reactivity recognize epitopes including highly conserved fusion peptide residues [19], [25], [43], [44], [46].

**Table 1:** Nucleotide sequences of mutagenic primers used and % VLP secretion from resultant plasmids relative to wild-type (100%).

Primer	Primer Sequence (5′-3′)^1^	Nucleotide Substitution	Amino Acid Substitution	% VLP Secretion^2^
G104E	TTTCCAAATAGTCCACA**T**TCATTTCCCCATCCTCTGTCTACC	GGA-GAA	Gly-Glu	∼0
G104H	CCCTTTCCAAAT6AGTCCACA**GTG**ATTTCCCCATCCTCTGTCTACC	GGA-CAC	Gly-His	∼0
G106M	GCCTCCCTTTCCAAATAG**CAT**ACATCCATTTCCCCATCC	GGA-ATG	Gly-Met	100
G106R	GCCTCCCTTTCCAAATAG**A**C**G**ACATCCATTTCCCCATCC	GGA-CGT	Gly-Arg	100
G106D	GCCTCCCTTTCCAAATAG**AT**CACATCCATTTCCCCATCC	GGA-GAT	Gly-Asp	150
G106W	GCCTCCCTTTCCAAATAG**C**C**A**ACATCCATTTCCCCATCC	GGA-TGG	Gly-Trp	<50
G106Q	GCCTCCCTTTCCAAATAGT**TG**ACATCCATTTCCCCA	GGA-CAA	Gly-Gln	150
L107Q	GCCTCCCTTTCCAAAT**TC**TCCACATCCATTTCCCCATCC	CTA-GAA	Leu-Gln	150
L107D	GCCTCCCTTTCCAAA**ATC**TCCACATCCATTTCCCCATCC	CTA-GAT	Leu-Asp	<50
L107M	GCCTCCCTTTCCAAA**C**A**T**TCCACATCCATTTCCCCATCC	CTA-ATG	Leu-Met	50
L107G	GCCTCCCTTTCCAAA**ACC**TCCACATCCATTTCCCCATCC	CTA-GGT	Leu-Gly	100
L107K	GGTCACAATGCCTCCCTTTCCAAAT**TT**TCCACATCCATTTCCCC	CTA-AAA	Leu-Lys	75
L107F	GCCTCCCTTTCCAAA**A**A**A**TCCACATCCATTTCCCCATCC	CTA-TTT	Leu-Phe	150
G106D/L107K	CACAATGCCTCCCTTTCCAAAT**TTGT**CACATCCATTTCCCCATCC	GGA-GAC CTA-AAA	Gly-Asp Leu-Lys	67
G106D/L107D	CACAATGCCTCCCTTTCCAAA**GTCGT**CACATCCATTTCCCCATCC	GGA-GAC CTA-GAC	Gly-Asp Leu-Asp	<5
G106R/L107D	CACAATGCCTCCCTTTCCAAA**GTCG**C**G**ACATCCATTTCCCCATCC	GGA-CGC CTA-GAC	Gly-Arg Leu-Asp	8
G106R/L107K	CACAATGCCTCCCTTTCCAAA**CTTG**C**G**ACATCCATTTCCCCATCC	GGA-CGC CTA-AAG	Gly-Arg Leu-Lys	85
G106W/L107K	CACAATGCCTCCCTTTCCAAA**CTTC**C**A**ACATCCATTTCCCCATCC	GGA-TGG CTA-AAG	Gly-Trp Leu-Lys	17
G106W/L107D	CACAATGCCTCCCTTTCCAAA**GTCC**C**A**ACATCCATTTCCCCATCC	GGA-TGG CTA-GAC	Gly-Trp Leu-Asp	67
K305E	TATTTCCTTCACAACTTTAAACT**C**TCCTGTGCACATAGAG	AAG-GAG	Lys-Glu	6
K305N	TATTTCCTTCACAACTTTAAA**G**TTTCCTGTGCACATAGAG	AAG-AAC	Lys-Asn	13
V308N	CCATGTTGTGTTTCTGCTATTTCCTTCACA**TT**TTTAAACTTTCCTGT	GTT-AAT	Val-Asn	19
K310E	TGTGTTTCTGCTATTTCCT**C**CACAACTTTAAACTTTCCTGT	AAG-GAG	Lys-Glu	25
K310Q	TGTGTTTCTGCTATTTCCT**G**CACAACTTTAAACTTTCCTGT	AAG-CAG	Lys-Gln	38
E311R	CCATGTTGTGTTTCTGCTATT**CG**CTTCACAACTTTAAACTTTCCTG	GAA-CGA	Glu-Arg	100
E311K	CCATGTTGTGTTTCTGCTATTT**T**CTTCACAACTTTAAACTTTCCTG	GAA-AAA	Glu-Lys	38
E311Y	CCATGTTGTGTTTCTGCTAT**G**T**A**CTTCACAACTTTAAACTTTCCTG	GAA-TAC	Glu-Tyr	6
K310E/E311R	GTGTTTCTGCTATT**CG**CT**C**CACAACTTTAAACTTTCCTGTGC	AAG-GAG GAA-GGA	Lys-Glu Glu-Arg	25
R323E	CCGTCCCCTTCATATTGCACT**TC**GATAACTATTGTTCCAT	AGA-GAA	Arg-Glu	6
R323M	CCGTCCCCTTCATATTGCAC**CA**TGATAACTATTGTTCCAT	AGA-ATG	Arg-Met	17
K361D	TATGTTGACTGGGCTATC**A**T**C**TTCTGTCACAATTGGGTTG	AAA-GAT	Lys-Asp	38
P364R	AGGTTCTGCTTCTATGTTGAC**CC**GGCTATCTTTTTCTGTC	CCA-CGG	Pro-Arg	25
P364E	GGAGGTTCTGCTTCTATGTTGACT**TC**GCTATCTTTTTCTGTC	CCA-GGA	Pro-Glu	25
K388D	GCTTCCTTTCTTAAACCAGTTGAG**A**T**C**CAGTTGTCCCG	AAG-GAT	Lys-Asp	50
K388E	GCTTCCTTTCTTAAACCAGTTGAG**T**T**C**CAGTTGTCCCG	AAG-GAA	Lys-Glu	50

1 Mutated nucleotides are shown in bold.

2 Average of triplicate experiments of mutant VLP secretion from transiently transformed COS-1 cells, standardized against the wild-type DENV-2 plasmid VLP secretion.

**Table 2:** MAb reactivities for DENV-2 virus-like particle (VLP) mutants^1^.

**MAb:**		Rabbit^2^	MHIAF	4G2	6B6C-1	4A1B-9	23-1	23-2	20	5-1	5-2	1B7-5	D3-5C9-1	1A1D-2	9D12	10A4D-2	1B4C-2	3H5
**CR** 3 **:**		poly-clonal	poly-clonal	group	group	group	group	group	sub grp.	sub grp.	sub grp.	comp.	comp.	sub comp.	sub comp.	sub comp.	sub comp.	type-spec.
**Virus** 4 **:**		D2	D2	D2	SLEV	MVEV	WNV	JEV	D2	JEV	JEV	D3	D4	D2	D1	D2	D2	D2
	**Percent Secretion** 5																	
WT DENV-2^6^	100	5.0	6.2	≥6.0	≥6.0	5.1	≥6.0	≥6.0	≥6.0	≥6.0	≥6.0	≥6.0	5.1	≥6.0	≥6.0	≥6.0	4.5	≥6.0
G106R	100	nd	25	**<0.1**	**0.8**	**0.4**	100	100	100	100	100	25	100	25	25	100	**1.5**	100
L107D	<50	nd	100	**<0.1**	25	25	**<0.1**	25	100	100	100	100	100	50	100	100	**1.5**	100
G106R-L107D (EDII_FP_)	8	100	100	**<0.1**	**<0.1**	**<3**	**0.2**	**0.2**	100	**3**	100	100	25	100	100	**6**	**<3**	50
K305E	6	100	100	nd^7^	nd	nd	nd	nd	100	100	100	100	50	**3**	100	100	100	**<0.1**
K310E	25	100	100	nd	nd	nd	nd	nd	100	100	100	100	100	**<3**	**<3**	100	100	100
E311R	100	50	100	nd	nd	nd	nd	nd	**<0.1**	**<0.1**	100	100	50	**3**	100	100	12.5	100
P364R	25	100	100	nd	nd	nd	nd	nd	100	100	100	25	50	**6**	50	25	**2.5**	100
K388D	50	100	100	nd	nd	nd	nd	nd	100	100	100	100	100	**6**	100	100	50	100
K310E-E311R- P364R (EDIII_CR_)	19	100	100	100	100	150	100	100	**<0.1**	**<0.1**	100	100	100	**0.2**	**0.1**	100	200	100
G106R-L107D- K310E-E311R- P364R (EDII_FP_−EDIII_CR_)	33	100	100	**<0.1**	**0.2**	**<0.8**	**<0.1**	**0.2**	**<0.1**	**<0.1**	100	100	**1.5**	**0.1**	**0.4**	100	**1.6**	100

1 Reactivities levels of MAbs of varying cross-reactivity (CR) selected from different flaviviruses for wildtype (WT) and mutant VLP antigens.

2 Rabbit anti-DENV-2 VLP-immunized hyper-immune sera used as detector for antigens captured with MHIAF.

3 Antibody Cross-reactivity (CR): Rabbit anti-DENV-2 and murine hyper-immune ascitic fluid (MHIAF) are polyclonal; ‘group’ CR antibodies recognize viruses of the four major pathogenic flavivirus serocomplexes; ‘sub-group’ CR MAbs recognize all or some members of two or more different flavivirus serocomplexes (e.g., MAbs 20, 5-1 and 5-2 recognize DENV-2 and JEV, JEV and DENV-2 and JEV, DENV-1 and DENV-2 respectively); ‘comp.’ and ‘sub-comp.’ CR MAbs recognize all four DENV complex viruses or a subset thereof respectively, and type MAbs recognize only DENV-2.

4 Virus the MAb was raised against; D1 = dengue virus serotype-1 (DENV-1), D2 = DENV-2, D3 = DENV-3, D4 = DENV-4, SLEV = St. Louis encephalitis virus, MVEV = Murray Valley encephalitis virus, WNV = West Nile virus, and JEV = Japanese encephalitis virus.

5 Percent secretion of mutant VLP constructs relative to WT. All values are the average of three independent experiments.

6 MAb reactivities for WT DENV-2 VLP are presented as inverse log_10_ Ag-capture ELISA endpoint values and mutant VLPs as the percent remaining reactivity compared to WT. Emboldened values represent reactivity reductions greater than 90% relative to WT.

7 nd denotes not determined.

**Figure 2:**
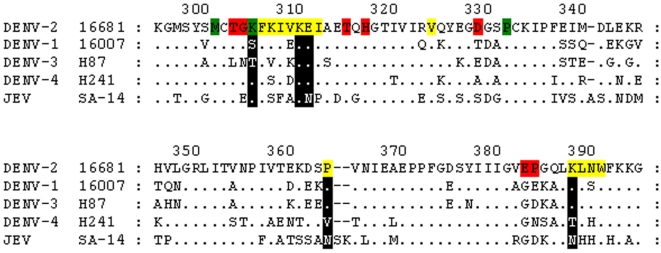
Envelope protein structural domain III (EDIII) alignment of representative strains of the four dengue virus (DENV) serotypes and Japanese encephalitis virus (JEV). Single letter amino acid abbreviations are shown for EDIII of DENV-2 using DENV-2 numbering (the last digit of the residue number lies directly above the numbered residue). Amino acids conserved relative to DENV-2 in the other serotypes are shown as dots, alignment gaps are depicted with dashes, and single letter abbreviations for non-conserved amino acids are shown. Colored residues in the DENV-2 sequence depict epitope-specific determinates as determined in this report and previously published reports. DENV complex and subcomplex cross-reactive epitopes are highlighted in yellow, DENV-2 specific residues are highlighted in red, and residues from the region of overlap between these epitopes (hence affecting DENV complex and DENV-2 virus specific epitopes) are highlighted green [22], [23], [24], [47]. The substituted EDIII residues incorporated into mutant antigens in this study are marked in black for the non DENV-2 viruses: DENV EDIII complex cross-reactive knock out mutants incorporated K310D, E311R, P364R, and K388D; K305E was utilized to determine EDIII DENV-2 specific immunoglobulin responses.

We next examined the effects of 15 amino acid substitutions at eight different residues in EDIII, first alone and then in combination (Table 1). The EDIII substitutions examined primarily altered the reactivity of DENV complex MAbs (Table 2). The residues that had the greatest effects on MAb reactivities were K305, K310, E311 and P364 (Fig. 1, 2 and Table 2). The MAbs affected by substitutions at these four residues exhibited three different reactivity patterns: those recognizing some but not all viruses in both the JEV and DENV serocomplexes (MAbs 20 and 5-1), MAbs recognizing only viruses within the DENV complex (1A1D-2, 9D12, 10A4D-2, and 1B4C-2) and DENV-2 type-specific MAbs (3H5). The K305 substitutions (K305E and K305N) were the only EDIII substitutions that significantly affected DENV-2 type-specific antibody recognition. K305 substitutions also affected DENV complex MAb 1A1D-2. There were no significant effects of the K305 substitutions for other DENV complex MAbs.

Some of the same antibodies exhibiting reactivity reductions from fusion peptide substitutions were also affected by EDIII substitutions (Table 2). For example, MAb 5-1 exhibited reactivity reductions to all six fusion peptide double mutant VLPs and also for one of three E311 substitutions. VLPs containing the charge reversal substitution E311R lost all measurable ELISA recognition by MAb 5-1 yet VLPs containing the alternative charge reversal substitution E311K or E311Y were unchanged. Similarly, DENV subcomplex MAb 10A4D-2 exhibited reactivity reductions for VLPs containing substitutions in either EDIII or the EDII fusion peptide. These results extend previous observations that some cross-reactive antibodies might recognize intra-E dimer epitopes spatially overlapping EDII and EDIII structural domains of independent monomers [44], [45].

The third class of antibodies recognizing epitope determinates in EDIII were DENV complex and subcomplex MAbs. The antibody panel contained six DENV complex cross-reactive MAbs, three raised from DENV-2 and one each from the other three serotypes. The two MAbs exhibiting the greatest decreases in EDIII reactivity were 1A1D-2 and 9D12, subcomplex reactive MAbs raised against DENV-2 and -1 respectively (Table 2). 1A1D-2 reactivity was reduced by single substitutions at all DIII residues investigated with the exception of K361, consistent with recent crystal structure data from the 1A1D-2 antigen-binding fragment complexed with recombinant DENV-2 EDIII (Tables 1 and 2;[47]). MAb 9D12 reactivity was only affected by the K310 and P364 individual substitutions. However, EDIII substitutions, both individual and in combination did not dramatically affect the reactivity of DENV complex reactive MAbs 1B7 or D3-5C9-1, antibodies raised against DENV-3 and -4 respectively (Table 2).

Substitutions from the EDII fusion peptide and from EDIII were combined into the same plasmid construct in an attempt to maximize reductions in cross-reactive antibody recognition of epitopes incorporating these two disparate structural regions. The general effect on MAb reactivities of combining substitutions was predictably additive. In general there was little effect of DIII substitutions on the group cross-reactive MAbs, and limited effect of fusion peptide substitutions on most complex and subcomplex MAbs. When substitutions were combined together the resulting VLPs exhibited reduced reactivities to both classes of antibodies (Table 2). We also observed significant reductions in the reactivity of DENV-4 derived complex cross-reactive MAb D3-5C9-1. By combining epitope determinates from both domains we were able to construct mutant VLPs exhibiting significantly reduced or ablated reactivity to the majority of MAbs in the panel representing five distinct reactivity classes: group, subgroup, complex and subcomplex cross-reactive, and DENV-2 specific antibodies (Table 2). Thus, this series of cross-reactive and DENV-2 specific epitope knock-out VLPs can be utilized as serodiagnostic antigens to characterize the epitope-specific targeting of the polyclonal human immunoglobulin response to DENV infection.

### Epitope-specific Humoral Immune Responses of DENV-2 Infected Patients

We selected a number of well-characterized DENV-2 infected patient sera to examine the epitope-specific proportions of the polyclonal E protein-specific immune response using the mutant VLP antigens described in this report. All serum specimens were collected 6–18 days post onset of symptoms (dpo) from DENV-2 infected individuals suffering from dengue fever and were viral RNA positive as determined by a DENV-2 specific reverse transcriptase-PCR (RT-PCR) assay (Table 3). Primary and secondary DENV-2 infected patient sera were selected from each of two different geographic regions; Puerto Rico and Taiwan (Table 3). IgM and IgG end-point titers were determined using six distinct DENV-2 VLP antigens to estimate the proportions of immunoglobulin recognizing four distinct E-protein antigenic regions (Table 2). Wild-type (WT) DENV-2 VLP was used to determine total anti-E IgM and IgG titers. Immunoglobulin proportions targeting EDII fusion peptide epitopes were determined with EDII fusion peptide knock-out mutant G106R-L107D (EDII_FP_). Immunoglobulin recognizing cross-reactive epitopes in EDIII was determined with the EDIII cross-reactive knock-out mutant K310E-E311R-P364R (EDIII_CR_) and total immunoglobulin proportions recognizing all cross-reactive epitopes incorporating determinates in either or both EDII fusion peptide and/or EDIII were determined with an EDII-III combination of these two knock-out mutants G106R-L107D-K310E-E311R-P364R (EDII_FP_−EDIII_CR_). The proportion of EDIII DENV-2 virus-specific immunoglobulin was determined as the difference between the EDIII K305E and K388D antigen reactivities (EDIII_TS_; Fig. 1c, 2, and Table 2). Endpoint titers of each serum specimen were determined concurrently in IgM and IgG antibody-capture ELISA with each DENV-2 antigen and a negative antigen. As described in the methods section, immunoglobulin endpoints were calculated for the different VLP antigens using asymptotic covariance matrix ***V*** method by regression analysis with four variables: antigen class, immunoglobulin class (IgM, IgG), primary or secondary infection status, and the resident country of infected patients (Puerto Rico or Taiwan); and a general least squares (GLS) approach was applied to determine endpoints. Because the mutant antigens are epitope knock-outs, the domain-associated, epitope specific immunoglobulin percentages were determined by dividing the endpoint titer determined with a knock-out antigen by the titer of the WT antigen, subtracting this value from 1.0 and multiplying by 100. DENV-2 virus specific imunoglobulin percentages were determined as the difference between the percent recognizing the K305E and the K388D antigens.

**Table 3:** Serological characterization of DENV-2 infected sera examined in this study.

Sera #	Country of Origin	DPO^1^	1°/2° 2	IgM P/N	IgG P/N	M/G OD-N^3^
4	Taiwan	17	1°	46.0	20.4	1.65
5	Taiwan	17	1°	27.8	10.3	2.53
12	Taiwan	14	1°	33.7	29.0	1.19
16	Taiwan	18	1°	5.02	3.59	1.25
8882	Puerto Rico	6	1°	44.2	5.04	11.1
0078	Puerto Rico	10	1°	65.0	12.1	4.23
9	Taiwan	14	2°	35.6	24.4	0.91
10	Taiwan	18	2°	15.6	24.1	0.41
17	Taiwan	14	2°	6.1	24	0.21
0169	Puerto Rico	6	2°	29.2	35.4	0.65
9608	Puerto Rico	13	2°	33.4	39.8	0.78
8867	Puerto Rico	7	2°	53.6	54.2	1.04

1 DPO denotes days post onset of symptoms.

2 denotes primary (1°) or secondary (2°) DENV-2 infection.

3 This column denotes the ratio of the optical density (OD) for IgM/divided by IgG. Each value is corrected by subtracting 2 times the negative OD value.

Results from ANOVA on the GLS regression indicated that the three-way interaction among antigen, immunoglobulin type and geographic origin was not statistically significant (p = 0.6810). This result suggests that the patterns observed in mean endpoints among the different antigens, within the same immunoglobulin class are similar for sera collected from two different geographic locations, Taiwan or Puerto Rico. Because this three-way interaction was not significant we reran the ANOVA examining only main and two-way interactions (Table 4). There is evidence that the two-way interactions between immunoglobulin type and primary or secondary infection status (p<0.0001), between antigen class and primary or secondary infection status (p<0.0001), between antigen class and geographic origin (p = 0.0403), and between antigen class and immunoglobulin type (p<0.0001) were statistically significant. There were highly significant effects of three of the four individual variables on epitope-specific endpoint titers (p<0.0001); the only individual variable not having a significant effect on antigen endpoints was the geographic origin of the sera (p = 0.0709). In other words, there were no differences in total endpoint titer averaged across the different antigens, between the DENV-2 infected patient sera from Puerto Rico and from Taiwan (Table 4). The significant interaction between antigen class and geography does however indicate that there could be significant geographical effects on the magnitudes of individual epitope-specific immunoglobulin populations. Such differences could arise either from differential immune status correlated with geography and/or from the different genetic backgrounds of patients from Taiwan and Puerto Rico.

**Table 4:** Analysis of variance table of calculated DENV-2 infected serum end-point data determined with different epitope specific antigens.

Variable	F-value	p-value
Antigen	370	<0.0001
Immunoglobulin type	239	<0.0001
1° or 2° Infection	362	<0.0001
Geographic origin of Sera	3.36	0.0709
Antigen : Immunoglobulin type	17.7	<0.0001
Antigen : Geographic origin of Sera	2.91	0.0403
Antigen : 1° or 2° Infection	30.8	<0.0001
Immunoglobulin type : 1° or 2° Infection	118	<0.0001

Because the ANOVA results suggest no generalized effect of geographic origin of test sera, the source of sera are not further specified unless indicated. Endpoint titers of E-protein specific immunoglobulins from DENV-2 infected patient sera (measured with the WT DENV-2 antigen) ranged from 21,400–1,510,000 (mean = 299,000) for IgM and from 12,400–11,000,000 for IgG (mean = 2,400,000; Table 5). Primary DENV-2 infected patient sera exhibited similar IgM and IgG titers for individuals and as a class these sera had IgM and IgG titers ranging from 21,400–1,510,000 (mean = 408,000) and 12,400–1,020,000 (mean = 376,000) respectively (Table 6). Secondary DENV-2 infected patient sera exhibited a distinctly different pattern with IgG titers ranging from seven to over 100 times greater than IgM titers within individual sera (21,900–447,000; mean = 189,000 for IgM and 2,290,000–11,000,000; mean = 4,430,000 for IgG; Table 6). The larger magnitude IgG response in secondary DENV infections could result from the rapid and strong memory B- and T-cell response and the increased frequency of reduced IgM response in secondary DENV infections [28]. Although all serum specimens were late acute to early convalescent phase (6–18 dpo) this is sufficient time for typically rapid and strong anamnestic IgG responses.

**Table 5:** Epitope Specific Proportions of Envelope Protein Specific IgM and IgG from DENV-2 Infected Human Sera.

	DENV-2 Antigen^1^	Epitope Specific Target^2^	Mean Endpoint Titer^3^	Endpoint Titer Range	Range of Percent Response^4^	Mean (Median) Percent Epitope Specific Response
***IgM***						
	WT	All	2.99×10^5^	2.14×10^4^–1.51×10^6^	100	100
	G106R-L107D*†	EDII_FP_	1.66×10^5^	6.07×10^2^–4.79×10^5^	<1–97	44 (34)
	K310E-E311R-P364R†	EDIII_CR_	1.71×10^5^	1.59×10^4^–8.32×10^5^	<1–90	43 (39)
	G106R-L107D-K310E-E311R-P364R*	EDII_FP_−EDIII_CR_	1.26×10^5^	5.00×10^2^–3.98×10^5^	<1–91	58 (67)
	K305E-K388D	EDIII_TS_	1.79×10^4^	2.19×10^2^–7.98×10^4^	<1–20	6 (4.5)
***IgG***						
	WT*†	All	2.40×10^6^	1.24×10^4^–1.10×10^7^	100	100
	G106R-L107D*	EDII_FP_	1.54×10^6^	7.37×10^2^–4.79×10^6^	<1–94	36 (36)
	K310E-E311R-P364R†	EDIII_CR_	2.46×10^6^	1.16×10^4^–1.05×10^7^	<1–26	5.8 (13)
	G106R-L107D-K310E-E311R-P364R	EDII_FP_−EDIII_CR_	6.76×10^5^	4.74×10^2^–1.70×10^6^	<1–96	72 (64)
	K305E-K388D	EDIII_TS_	2.40×10^4^	1.24×10^2^–4.50×10^5^	<1–8	1 (<1)

1 The mutant antigens used are all knock-out mutants. The symbols * and † depict significantly different mean endpoint titers determined with antigens sharing the same symbol. Mean endpoint titers were considered significantly different when the 95% confidence interval for the difference between the means did not cross zero.

2 Epitope specific antibody populations targeted by the different knock-out antigens. WT antigen measures antibody recognizing all E-protein epitopes. EDII_FP_ denotes broadly cross-reactive epitopes incorporating the E-protein structural domain II fusion peptide. EDIII_CR_ denotes predominately complex cross-reactive epitopes incorporating residues within E-protein structural domain III. EDII_FP_−EDIII_CR_ denotes individual or overlapping epitopes incorporating either or both the EDII fusion peptide or EDIII. EDIII_TS_ denotes EDIII DENV-2 type-specific epitopes and were determined by the reactivity difference between the K305E and K388D antigens (see methods for details).

3 Endpoint titers determined with the knock-out antigen, thus representing immunoglobulins recognizing epitopes not targeted by the knock-out antigen. Because the EDIII DENV-2 type-specific response was calculated as the percent difference between K305E and K388D reactivities, the titers for EDIII_TS_ were calculated as the WT titers multiplied by the percent EDIII_TS_ response.

4 Because the mutant antigens knock-out antibody recognition of specific epitopes, the percent of immunoglobulin recognizing a particular epitope was determined for each individual sera by calculating the percent reactivity measured with a mutant antigen relative to that determined with the WT antigen and subtracting this value from 1.0; (1- [Endpoint_mutant_/Endpoint_wt_])×100; for the percent DENV-2 specific reactivity we used (1-[(Endpoint_K305E_/Endpoint_WT_)−(Endpoint_K388D_)/Endpoint_WT_)])×100.

**Table 6:** Epitope Specific Proportions of Envelope Protein Specific IgM and IgG from Primary and Secondary DENV-2 Infected Human Sera.

	Infection Status	DENV-2 Antigen^1^	Epitope Specific Target^2^	Mean End-point Titer^3^	Endpoint Titer Range	Mean Percent Epitope Specific Response^4^
***IgM***						
	Primary	WT	All	4.08×10^5^	2.14×10^4^–1.51×10^6^	100
		G106R-L107D	EDII_FP_	1.89×10^5^	6.07×10^2^–4.79×10^5^	54
		K310E-E311R-P364R	EDIII_CR_	2.57×10^5^	1.59×10^4^–8.32×10^5^	37
		G106R-L107D-K310E-E311R-P364R	EDII_FP_−EDIII_CR_	1.53×10^5^	5.00×10^2^–3.47×10^5^	63
		K305E-K388D	EDIII_TS_	1.70×10^4^	1.07×10^3^–2.74×10^4^	4
	Secondary	WT*†	All	1.89×10^5^	2.19×10^4^–4.47×10^5^	100
		G106R-L107D*	EDII_FP_	1.44×10^5^	1.66×10^4^–4.07×10^5^	24
		K310E-E311R-P364R	EDIII_CR_	8.55×10^4^	1.95×10^4^–2.34×10^5^	55
		G106R-L107D-K310E-E311R-P364R†	EDII_FP_−EDIII_CR_	9.91×10^4^	1.32×10^4^–3.98×10^5^	48
		K305E-K388D	EDIII_TS_	1.48×10^4^	2.19×10^2^–3.66×10^4^	8
***IgG***						
	Primary	WT	All	3.76×10^5^	1.24×10^4^–1.02×10^6^	100
		G106R-L107D	EDII_FP_	2.10×10^5^	7.37×10^2^–6.92×10^5^	44
		K310E-E311R-P364R	EDIII_CR_	3.60×10^5^	1.16×10^4^–8.91×10^5^	7.5
		G106R-L107D-K310E-E311R-P364R	EDII_FP_−EDIII_CR_	1.68×10^5^	4.74×10^2^–4.90×10^5^	55
		K305E-K388D	EDIII_TS_	3.76×10^3^	1.24×10^2^–1.02×10^4^	<1
	Secondary	WT*†	All	4.43×10^6^	2.29×10^6^–1.10×10^7^	100
		G106R-L107D*†	EDII_FP_	2.87×10^6^	1.45×10^6^–4.79×10^6^	35
		K310E-E311R-P364R	EDIII_CR_	4.57×10^6^	2.00×10^6^–1.05×10^7^	4.0
		G106R-L107D-K310E-E311R-P364R	EDII_FP_−EDIII_CR_	1.18×10^6^	7.08×10^5^–1.70×10^6^	73
		K305E-K388D	EDIII_TS_	8.86×10^4^	2.29×10^4^–4.50×10^5^	2

1 The mutant antigens used are all knock-out mutants. The symbols * and † depict significantly different mean endpoint titers between those antigens sharing the same symbol. Mean endpoint titers were considered significantly different when the 95% CI for the difference between the mean endpoints did not cross zero.

2 Epitope specific antibody populations targeted by the different knock-out antigens. WT antigen measures antibody recognizing all E-protein epitopes. EDII_FP_ denotes broadly cross-reactive epitopes incorporating the E-protein structural domain II fusion peptide. EDIII_CR_ denotes predominately complex cross-reactive epitopes incorporating residues within E-protein structural domain III. EDII_FP_−EDIII_CR_ denotes individual or overlapping epitopes incorporating either or both the EDII fusion peptide or EDIII. EDIII_TS_ denotes EDIII DENV-2 type-specific epitopes and were determined by the reactivity difference between the K305E and K388D antigens (see methods for details).

3 Endpoint titer determined with the knock-out antigen, thus representing immunoglobulins recognizing epitopes not targeted by the knock-out antigen. Because the EDIII DENV-2 type-specific response was calculated as the percent difference between K305E and K388D reactivities, the titers for EDIII_TS_ were calculated as the WT titers multiplied by the percent EDIII_TS_ response.

4 Because the mutant antigens knock-out antibody recognition of specific epitopes, the percent of immunoglobulin recognizing a particular epitope was determined for each individual sera by calculating the percent reactivity measured with a mutant antigen relative to that determined with the WT antigen and subtracting this value from 1.0; (1- [Endpoint_mutant_/Endpoint_wt_])×100; for the percent DENV-2 specific reactivity we used (1-[(Endpoint_K305E_/Endpoint_WT_)−(Endpoint_K388D_)/Endpoint_WT_)])×100.

### Cross-reactive immunoglobulin populations from two overlapping antigenic regions form the majority of the immune response and are greater in magnitude for IgM than for IgG

Immunoglobulin proportions targeting cross-reactive EDII fusion peptide epitopes were highly variable yet tended to be large, and were distinctly greater in primary than in secondary DENV-2 infected patients. EDII fusion peptide specific immunoglobulin responses ranged from undetectable levels to 97% of IgM and from undetectable to 94% of IgG; the mean and median values were 44% and 34% of IgM and 36% and 36% of IgG respectively (Table 5). The larger magnitude fusion peptide-specific immunoglobulin response in primary versus secondary DENV-2 infections was greater in IgM than in IgG (54% vs. 24% and 44% vs. 35% in IgM and IgG respectively; Table 6). EDII fusion peptide-specific IgM comprised a greater percentage of the total IgM response for the DENV-2 infected patient sera from Taiwan (51%) than it did for the Puerto Rican sera (33%). This was the only epitope-specific immunoglobulin subclass where there was a dramatic percent difference between serum specimens from the two geographic regions. The significance of these differences is supported by the ANOVA results: although there was non-significant trend towards an effect of geographic origin of the sera alone on the total immunoglobulin titers (p = 0.0709) there was a significant two-way interaction between epitope-specific antigen classes and the geographic origin of the sera on these end-points (p = 0.0403; Table 4).

Immunoglobulin targeting cross-reactive EDIII epitopes showed similar trends as did that recognizing EDII fusion peptide epitopes but their variability and magnitudes were smaller and there was an even greater reduction in IgG titers relative to IgM. EDIII cross-reactive immunoglobulin ranged from <1% to 45% in primary infections and <1% to 90% in secondary infections. IgM recognizing cross-reactive EDIII epitopes averaged 37% and 55% in primary and secondary infections respectively, whereas EDIII cross-reactive IgG averaged only 7.5% and 4% of primary and secondary DENV-2 infected patient sera (Table 6). Thus, there was a much greater EDIII cross-reactive IgM response than in IgG and for IgM this response was greater in secondary than in primary infections.

In addition to the EDII fusion peptide and the EDIII cross-reactive epitope knock-out antigens, serum specimens were screened with an EDII−EDIII knock-out antigen, combining the same substitutions from the individual mutant antigens in these two antigenic regions (EDII_FP_−EDIII_CR_; Table 2). Immunoglobulin recognizing epitopes in either of -or overlapping- these structural domain regions averaged 58% of IgM and 72% of the IgG present (median = 67% and 64% respectively; Table 5). There was a greater total cross-reactive IgM response in primary versus secondary DENV-2 infected sera (63% and 48% respectively). However, this pattern was reversed in IgG where 55% and 73% recognized these cross-reactive epitopes in primary and secondary DENV-2 infected sera respectively (Table 6). Interestingly, the percent cross-reactive immunoglobulin as determined with the EDII_FP_−EDIII_CR_ antigen was not the same as estimated by the adding the percents of immunoglobulin recognizing the EDII fusion peptide and cross-reactive EDIII epitopes (Tables 5 and 6). There were exceptions to this general observation for IgM or IgG for some individual serum specimens. The total proportion of cross-reactive immunoglobulin determined with the EDII_FP_−EDIII_CR_ antigen was greater than that estimated by adding the percentages determined with the EDII_FP_ and EDIII_CR_ antigens for IgG (72% and 42% respectively), yet the opposite pattern was observed for IgM (EDII_FP_−EDIII_CR_ = 58%; EDII_FP_+EDIII_CR_ = 87%; Table 5). These data suggest that there are different patterns of E-protein antigenicity between IgM and IgG. In the MAb screening we observed significant reactivity reductions with DENV complex cross-reactive MAb D3-5C9-1 for the inter-domain EDII_FP_−EDIII_CR_ antigen, yet no reactivity reductions of this MAb for either the EDII_FP_ or the EDIII_CR_ antigens alone (Table 2). Thus, a proportion of human IgG might recognize epitopes similar to murine MAb D3-5C9-1 or they might recognize epitopes not represented in our MAb panel that similarly exhibit reduced recognition for the EDII_FP_−EDIII_CR_ antigen and not for either the EDII_FP_ or EDIII_CR_ antigens alone.

### Immunoglobulin populations targeting EDIII DENV-2 specific epitopes are small yet of greater magnitude in IgM than in IgG and in secondary than in primary DENV-2 infected sera

To determine the proportion of immunoglobulin recognizing EDIII DENV-2 type-specific epitopes identified as potently neutralizing in murine studies we used two different EDIII mutant antigens. K305E knocks out the reactivity of MAb 3H5 (Table 2) the prototype DENV-2 type-specific MAb recognizing this epitope. K305 has been demonstrated to be essential for the binding of MAb 3H5 and six other potently neutralizing DENV-2 specific MAbs [23]. However, K305E also reduced the reactivity of subcomplex cross-reactive neutralizing MAb 1A1D-2 (Table 2). The K388D antigen reduced only the reactivity of MAb 1A1D-2 - to similar levels as did K305E and both of these residues have been identified as 1A1D-2 binding residues in a recent DENV-2 structural determination with this MAb [47]. The amount of EDIII DENV-2 specific immunoglobulin was therefore determined as the difference in the percent reduction in endpoint titer relative to WT, between the K305E and K388D antigens.

EDIII DENV-2 virus-specific immunoglobulin formed very small percentages of the antibody response and was greater in IgM than in IgG. In contrast to the cross-reactive immunoglobulins however, EDIII virus-specific antibody populations averaged twice as large in secondary as in primary DENV-2 infected patient sera (Tables 5 and 6). EDIII DENV-2 specific IgM averaged 6%, was 4% and 8% in primary and secondary DENV-2 infections, and ranged from <1% to 20%. EDIII DENV-2 specific IgG averaged only 1% across all sera tested, was <1% and 2% in primary and secondary serum samples, and ranged from <1% to 8% of total IgG. Interestingly, serum specimens with the highest percent EDIII DENV-2 specific IgM response were not the same as those with the highest percent IgG targeting this epitope.

### Neutralizing antibody responses of primary and secondary DENV-2 infected patient sera: EDIII DENV-2 specific IgG titer is correlated with DENV-2 neutralization

Focus-reduction micro-neutralization (FRμNT) assays were conducted with the sera to see if homologous/heterologous virus neutralization correlated with differential percentages or titers of virus-specific or cross-reactive immunoglobulin populations. All sera were tested against five different viruses, DENV-1, -2, -3, -4, and JEV; the Puerto Rican sera were additionally screened against WNV. Serum samples were diluted two-fold from 1∶50 until the last positive dilution representing the 90% FRμNT titer was reached. Actual 90% endpoint titers were also calculated by a non-linear regression of the FRμNT data using a variable slope sigmoidal dose-response formula. The 90% neutralization titers of primary DENV-2 infected patient sera ranged from 1∶200 to 1∶800 and all were greater than or equal to four-fold higher than the next highest virus titer (Table 7). Secondary DENV-2 infected patient sera had much higher DENV-2 90% neutralization titers ranging from 1∶400 to 1∶6400 (calculated 90% range: 431 to 11,174, Table 8). Only two of the secondary DENV-infected sera had DENV-2 neutralization titers four-fold or greater than of other DENV serotypes (sera #0169 and #8867). Puerto Rico #9608 and Taiwan #9 each had equal 90% neutralization titers to another serotype, DENV-4 and DENV-1 respectively. Taiwan serum specimens #10 and #17 each had 90% neutralization titers four-fold and two-fold higher respectively for DENV-1 than for the currently infecting DENV-2, (Table 8). The period of neutralizing antibody dominance to the primary infecting virus after secondary infection with a heterologous DENV serotype has been shown to vary from less than a week to multiple weeks, so presumably these four serum specimens, collected 13–18 dpo have therefore not switched or are in the process of switching to DENV-2 dominance [28]. Most patient sera had JEV 90% neutralization titers <50, however, secondary DENV-2 infected serum samples #9 and #10 had calculated JEV 90% neutralization titers of 83 and 103 respectively. This was not surprising for these two Taiwanese sera since they were also IgG ELISA positive against JEV VLP antigen [25]. We were surprised that the three secondary DENV-2 infected patient sera from Puerto Rico exhibited positive 90% neutralization titers against JEV, greater than expected from DENV infected cross-reactivity. Because the Puerto Rico sera were collected in 2007 when WNV was known to be circulating on the island we decided post hoc to test all of the Puerto Rico sera for neutralizing antibodies against WNV. The three secondary DENV-2 infected specimens had calculated 90% neutralization titers against WNV of 122, 74, and 59. The primary DENV-2 specimens from Puerto Rico had calculated 90% neutralization titers of <15 for both JEV and WNV. Three primary DENV-2 infected sera from Taiwan had calculated JEV 90% neutralization titers <8 and one was 18 (Table 8). Taken together these results suggest that there is limited cross-neutralization from infection by viruses in the DENV complex to viruses in the JEV serocomplex. Thus, for at least one, and possibly all three of the secondary DENV-2 infected sera from Puerto Rico, the presence of JEV neutralizing antibody is consistent with JEV complex-cross-reactivity occurring from actual WNV exposure. We did not observe any significant correlations between the proportions or the total magnitude of cross-reactive immunoglobulin populations and heterologous neutralization to other DENV serotypes or to JEV or WNV, implying that the large cross-reactive antibody populations were either weakly or non-neutralizing or that there was large variation in the neutralizing capabilities of these immunoglobulins between serum specimens.

**Table 7:** Virus neutralization titers for primary DENV-2 infected serum samples from dengue fever patients.

Serum #	Country of Origin	DPO^1^	Virus	90% FRμNT titer^2^	Calculated 90% FRμNT Titer^3^
4	Taiwan	17	DENV-1	<50	17
			DENV-2	400	373
			DENV-3	<50	18
			DENV-4	<50	29
			JEV	<50	7
5	Taiwan	17	DENV-1	<50	3
			DENV-2	800	706
			DENV-3	<50	10
			DENV-4	<50	16
			JEV	<50	4
12	Taiwan	14	DENV-1	50	56
			DENV-2	400	603
			DENV-3	50	50
			DENV-4	<50	45
			JEV	<50	18
16	Taiwan	18	DENV-1	<50	4
			DENV-2	400	411
			DENV-3	<50	7
			DENV-4	<50	13
			JEV	<50	3
8882	Puerto Rico	6	DENV-1	<50	9
			DENV-2	200	144
			DENV-3	<50	15
			DENV-4	<50	24
			WNV	<50	4
			JEV	<50	3
0078	Puerto Rico	10	DENV-1	<50	16
			DENV-2	200	145
			DENV-3	<50	31
			DENV-4	<50	64
			WNV	<50	12
			JEV	<50	3

1 days post onset of symptoms.

2 Last positive titer in 90% Focus-reduction micro-neutralization (FRμNT) assay.

3 Calculated actual 90% neutralization titers based on a nonlinear regression of the FRμNT data using a variable slope sigmoidal dose-response model.

**Table 8:** Virus neutralization titers for secondary DENV-2 infected serum samples from dengue fever patients.

Serum #	Country of Origin	DPO^1^	Virus	90% FRμNT titer^2^	Calculated 90% FRμNT Titer^3^
9	Taiwan	14	DENV-1	6400	7378
			DENV-2	6400	11,174
			DENV-3	800	2376
			DENV-4	800	589
			JEV	<50	83
10	Taiwan	18	DENV-1	25600	25,334
			DENV-2	6400	6743
			DENV-3	320	3024
			DENV-4	800	991
			JEV	50	103
17	Taiwan	14	DENV-1	6400	4179
			DENV-2	3200	2076
			DENV-3	3200	2898
			DENV-4	800	915
			JEV	<50	3
0169	Puerto Rico	6	DENV-1	200	306
			DENV-2	3200	3821
			DENV-3	800	934
			DENV-4	200	167
			WNV	<50	59
			JEV	<50	17
9608	Puerto Rico	13	DENV-1	50	79
			DENV-2	400	431
			DENV-3	100	95
			DENV-4	400	526
			WNV	<50	74
			JEV	<50	25
8867	Puerto Rico	7	DENV-1	200	366
			DENV-2	3200	3177
			DENV-3	100	135
			DENV-4	800	1341
			WNV	50	122
			JEV	<50	38

1 days post onset of symptoms.

2 Last positive titer in 90% Focus-reduction micro-neutralization (FRμNT) assay.

3 Calculated actual 90% neutralization titers based on a nonlinear regression of the FRμNT data using a variable slope sigmoidal dose-response model.

**Figure 3:**
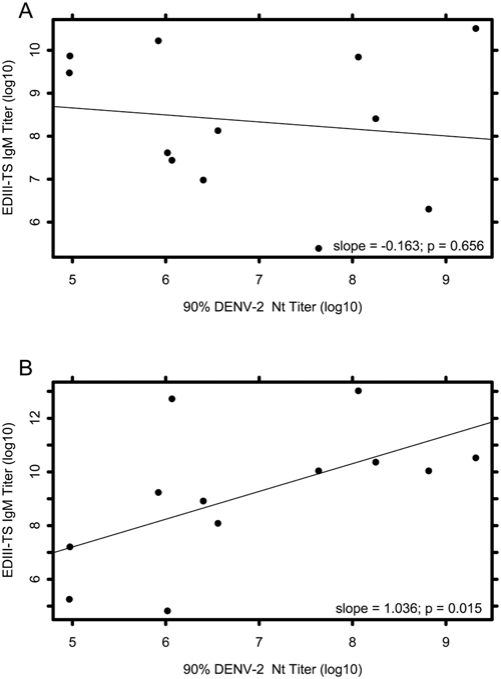
Envelope protein domain III DENV-2 specific (EDIII_TS_) IgG is positively and significantly correlated with DENV-2 neutralization. Log10 EDIII_TS_ IgM and IgG regressed on Log10 DENV-2 specific 90% neutralization endpoint titers. (A) EDIII_TS_ IgM titer is not associated with DENV-2 specific 90% neutralization titers (m = −0.163, p = 0.656), P value was determined by performing an analysis of variance on the slope of the regression. (B) EDIII_TS_ IgG is positively and significantly associated with increasing DENV-2 specific 90% Neutralization titers (m = 1.036, p = 0.0149), P value determined as in A.

To investigate if EDIII virus specific epitopes stimulate strongly neutralizing and protective immunoglobulin in humans as they do in mice we regressed EDIII DENV-2-specific (EDIII_TS_) IgM or IgG titers (log10, y-axis) on the actual DENV-2 90% neutralization titers (log 10) and performed an analysis of variance on the resulting regression (Fig. 3). For IgM, the regression slope was actually negative and not significantly different from 0 (m = −0.163, p = 0.656), suggesting that IgM recognizing EDIII_TS_ epitopes in these sera is not strongly neutralizing. However, when EDIII_TS_ IgG titers were regressed on DENV-2 neutralization, the slope was positive and significant (m = 1.036, p = 0.0149) indicating that increasing EDIII DENV-2 specific IgG is correlated with increasing neutralization (Fig. 3). These data suggest that the very small proportion of EDIII DENV-2 specific IgG is responsible for a significant proportion of DENV-2 specific neutralization, and that the potently neutralizing EDIII virus-specific epitopes identified in mice also play a role in DENV-2 specific neutralization in humans.

## Discussion

### MAb Epitope Mapping

Previous studies have indicated that the EDII fusion peptide region contains multiple, overlapping, broadly cross-reactive, immunodominant epitopes and that both virus-specific and DENV complex cross-reactive epitopes are located in EDIII [18], [19], [20], [21], [22], [23], [24], [25], [43], [44], [45], [46], [48]. The mutagenesis-based epitope mapping presented here supports and extends these results. Substitutions at either G106 or L107 ablate most group cross-reactive MAb recognition. This pattern was upheld in the current study with the exception of L107F which did not significantly reduce the binding of any group cross-reactive MAbs (data not shown). However, in JEV, WNV, SLEV, and tick-borne encephalitis virus (TBEV) E proteins the L107F substitution has been shown to reduce cross-reactive antibody recognition, and L107F TBEV recombinant subviral particles have been shown to reduce immunoglobulin recognition in polyclonal DENV infected human serum [14], [19], [43], [44]. In spite of the invariant nature of most fusion peptide residues, L107F does occur in a number of flaviviruses: Powassan and Deer Tick viruses, JEV vaccine strain SA-14-14-2, and DENV-2 strain POU-280 [49], [50], [51].

One of the more novel findings of the epitope mapping is that substitutions in different structural domains of adjacent E-protein monomers can act as epitope determinates for a single antibody suggesting that some antibodies might recognize inter-monomer epitopes including the disparate structural domains EDII and EDIII. Perhaps the best example of this was DENV-4 derived DENV complex cross-reactive MAb D3-5C9-1. This MAb exhibited only minor reactivity reductions (50–25%) for all six G106/L107 fusion peptide mutants and for two individual EDIII mutants E311R and P364R (50%). However, when the EDII fusion peptide and EDIII substitutions were combined into a single VLP construct, D3-5C9-1 reactivity was reduced to only 1.5% of WT DENV-2 reactivity. Such synergistic effects of combining substitutions on antibody reactivity could occur when residues do not play a critical binding role within the antibody paratope and thus the introduction of single substitutions might not severely interfere with antibody binding, yet when two or more substitutions of little to no individual effect are combined together, the combination either decreases K_a_ or increases K_d_ to the point where a significant reduction in antibody recognition is observed. Other examples of EDII−EDIII inter-monomer epitope determinates occurred with DENV subcomplex MAb 1B4C-2 and subgroup cross-reactive MAbs 5-1, and 5-2. MAb 1B4C-2 is a DENV-2 derived non-neutralizing antibody recognizing a surface accessible epitope originally assigned to EDI [18]. A previously published mutagenesis study demonstrated that EDII fusion peptide residues could act as epitope determinates affecting the binding of this MAb [20], and this study confirms the effect of both fusion peptide substitutions and EDIII substitutions acting as 1B4C-2 epitope determinates. Similar to MAb D3-5C9-1, the greatest decreases in binding of 1B4C-2 occurred for VLPs combining substitutions from both EDII and EDIII (Table 2).

MAb 5-1 is a JEV derived antibody that recognizes only JEV and DENV-2. All VLPs containing G106/L107 substitutions exhibited reduced binding by this MAb, as did E311R VLPs. Again, combinations of the EDII substitutions with EDIII E311R ablated all measurable MAb recognition for this VLP. MAb 20 is similar to MAb 5-1; it was raised against DENV-2 and also recognizes only JEV and DENV-2. Although fusion peptide substitutions did not alter the binding of this MAb, just as with MAb 5-1 all mutant VLPs containing combinations of E311 substitutions exhibited significant reductions in MAb 20 binding. E311 is conserved across the DENV complex and is an asparagine in the JEV complex viruses (Fig. 2); adjacent residue K310 is almost completely conserved across the mosquito borne flaviviruses, however K310 substitutions did not alter the binding of either MAb 20 or 5-1. Thus other shared residues between DENV-2 and JEV, possibly including fusion peptide residues, must be responsible for the identical cross-reactivity patterns of these two antibodies. MAb 5-2 is another JEV-derived subgroup cross-reactive MAb, recognizing JEV, DENV-1 and DENV-2. This study did not identify any substitutions in DENV-2 VLPs that disrupted the binding of this MAb. However, in JEV and in DENV-1, substitutions in both the fusion peptide and in EDIII significantly reduce the binding of this MAb [25].

The only other published report of similar inter-monomer EDII−EDIII epitopes is for chimpanzee MAb 1A5, a flavivirus group cross-reactive MAb raised against mixed infection of all four DENV serotypes [45]. G106 and H317 were independently identified from neutralization escape variants of MAb 1A5 and thus identified as epitope determinates. G106V significantly reduced antibody binding and neutralization whereas H317Q did not affect binding but did reduce neutralization by this MAb. Although structurally close to G106 (∼15Å) H317 lies buried on the bottom membrane surface of the E dimer in native virions, consistent with its lack of effect on 1A5 binding. The EDIII residue we identified as cooperating with fusion peptide residues G106 and L107 as a cross-reactive epitope determinate was E311. E311 is highly exposed on the outer surface of the dimer and is only 6–9Å from EDII fusion peptide residues G106 and L107 of the alternate monomer, well within the binding area of a typical Fab footprint (Fig. 1). Previously published competitive binding assays with DENV-2 identified an overlapping epitope cluster including MAbs recognizing fusion peptide epitopes such as 6B6C-1, EDIII epitopes such as 1A1D-2 and also MAb 1B4C-2, identified as recognizing an EDII−EDIII inter-domain epitope in this study [18]. These observations suggest that such inter-domain epitopes could be more common than previously recognized. It has been noted in this report and elsewhere that there is a diversity of overlapping epitopes containing fusion peptide residues recognized by antibodies with variable patterns of cross-reactivity [43], [44]. Due to the size of an antibody Fab footprint, it is expected that such epitopes will include residues outside of, and less conserved than those in the fusion peptide itself and that binding to these variable residues must account for the different patterns of cross-reactivity observed for antibodies recognizing epitopes in this region. Thus, it is not unexpected that there are antibody epitopes incorporating EDII fusion peptide residues and residues within the structurally adjacent EDIII of the alternate monomer. These results and interpretations suggest a point of caution for studies attempting to map antibody epitopes using only recombinant EDIII (rEDIII) protein or soluble E-protein monomers.

Some of the general and specific epitope mapping results presented here are supported by recently published studies. A number of studies have mapped DENV-complex and sub-complex cross-reactive epitopes to the lateral surface of EDIII [15], [16], [22], [24], [47], [48]. MAb 1A1D-2 is a DENV-2 derived subcomplex cross-reactive antibody that recognizes DENV-1, -2, and -3 [18]. We identified six residues located on three different β-strands in EDIII acting as epitope determinates for this antibody. Four of these residues are in β-strand A (Lys305, Val308, Lys310, and Glu311), and one each in β−strand B (Arg323) and β−strand G (Lys388) [15]. The seventh residue identified as an epitope determinate for this MAb, Pro364 is the last residue of the loop just prior to β−strand E. Recently published analyses using yeast surface display mutagenesis of DENV-2 rEDIII also identified MAb 1A1D-2 epitope determinates in β−strand A (Gly304, Lys305, Lys 307, and Lys310; [22]. In fact, a crystal structure of MAb 1A1D-2 Fab complexed with rEDIII was recently solved and confirms the principle involvement of EDIII β−strand A in 1A1D-2 binding [47]. Another recent study examined five different DENV subcomplex MAbs with the same cross-reactivity profiles as 1A1D-2. All five of these MAbs recognized a single antigenic site including residues in β−strands A, B, and G and centered on K310 [24]. It therefore appears that the well-characterized epitope recognized by MAb 1A1D-2 could be representative of most subcomplex antibodies recognizing DENV-1, -2, and -3.

MAb 9D12 was raised against DENV-1 and recognizes DENV-1, -2, and -4. This MAb appears to recognize an overlapping, yet distinct epitope from MAb 1A1D-2. Two of the residues we identified as epitope determinates for 1A1D-2 had similar effects on 9D12 reactivity; K310 and P364. Yeast display mutagenesis of rEDIII also identified the involvement of K310, but they did not examine the effect of substitutions at P364 for either 1A1D-2 or 9D12 binding [22]. There was one direct conflict between the 9D12 results presented here and those from a previous study. In this report there was no effect of the substitutions examined at K305 on 9D12 reactivity, whereas a previous study using rEDIII found significant reductions in 9D12 binding for a K305E mutant [22]. One possibility is that there could be subtle differences in EDIII conformational structure between VLPs and rEDIII. For example, there could be differences in local dynamic motions, and hence minor conformational perturbations of the K305E substitution when introduced into rEDIII compared to E protein dimers as displayed on the VLP surface. Such local dynamic motions have recently been observed in a recombinant EDIII system and demonstrated to be associated with nearby substitutions that correlate with reductions in MAb recognition and neutralization [52]. DENV complex cross-reactive MAb 4E11, which we did not examine, has been studied extensively in DENV-1 and also binds to a discontinuous EDIII epitope centered primarily around four residues in β−strand A, including K310 [48].

DENV type-specific antibodies, specifically the potently neutralizing murine MAbs, recognize epitopes in DIII [17], [18], [22], [23], [53], [54], [55], [56]. Although we purposefully selected amino acids that were likely to be incorporated into cross-reactive epitopes for this study we identified one residue, K305 that reduced the binding of the DENV-2-specific neutralizing MAb 3H5. 3H5 appears to be typical of many DENV-2 specific neutralizing MAbs, recognizing similar overlapping epitopes centered around EDIII residues K305 and P384 [22], [23]. Interestingly, in the same rEDIII yeast surface display mutagenesis study, P384 was identified as an important DENV-2 specific epitope determinate, yet not K305; despite the use of MAb 3H5 and K305 mutants in both of the previous and in this current study [22]. Sukupolvi-Petty et al. identified G304 as a major DENV-2 specific neutralizing MAb epitope determinate, G304 substitutions were not examined in this study or by Gromowski and Barrett and given its close proximity to K305, G304 could be included as a part of this epitope [22], [23]. A clear consensus result from all of these studies is that there exist two different overlapping antigenic regions on the lateral surface of EDIII, one stimulating antibody with varying levels of cross-reactivity in the DENV serocomplex and the other stimulatingDENV-2 virus-specific antibodies (Fig. 2). These results all stem from studies of murine MAbs and a major issue to be addressed is weather the outer lateral surface of DIII contains protective virus-specific neutralizing epitope determinates for humans as well [57].

### Characterization of DENV-2 Infected Patient's Serum

Recently there has been increased interest to examine and dissect complex polyclonal human immune responses to flavivirus infection. The results presented in this study compliment and add to this nascent body of work with detailed epitope-specific antibody assignments for IgM in addition to IgG. Stiasny et al. measured total E-specific IgG titers from six DENV-2 infected patient sera that ranged from 10^4^ to 10^6^ [19]; similar magnitudes and variation as determined in this study. Another recent study of WNV epitope-specific immune responses found much smaller and less variable E-specific IgG titers in WNV-infected patient sera (10^3^–10^4^) [26]. These WNV-infected sera were collected 4–7 months post onset of symptoms although a few sera collected less than one month post onset had similar IgG titers. The DENV-2 infected sera examined by Stiasny et al had low to no IgM, consistent with their also being convalescent phase sera. These observations suggest that the different magnitudes of E-specific IgG between DENV-2 and WNV infected patient sera in these studies could be a virus specific phenomenon. DENV viremia in humans lasts approximately 4–10 days whereas WNV viremia is more transient, lasting only 1–3 days. Thus, the larger magnitude IgG response in DENV-2 infected patients compared to WNV-infected patients could result from differences in the length of viremia between these two viruses. Clearly, more studies will be needed to determine if this is a general phenomenon. In this study, E-specific IgM titers for primary and secondary infected sera were also in the 10^4^–10^6^ range (Table 5), although IgM titers were not presented in the WNV study cited above, they would be expected to be low based on the late convalescent timing of the serum collections.

The importance of the EDII fusion peptide as an immunodominant antigenic region containing a series of overlapping epitopes stimulating broadly cross-reactive antibodies has been well-established in mice [19], [20], [26], [43], [44]. The results presented in this report and other recent studies confirm the extension of this observation to humans as well [19], [26], [58]. In this study IgG recognizing EDII fusion peptide epitopes ranged from undetectable to 94% of the E-specific IgG response and averaged 36% (Table 5). Stiasny et al also found variable levels of EDII fusion peptide recognizing IgG in DENV-2 infected patient sera, they did not quantify this variation but estimated the proportion of IgG recognizing these epitopes to average about 30% and confirmed the limited neutralizing capability of this antibody population [19]. Because their study examined convalescent phase sera, the similar percentage estimates of IgG targeting EDII fusion peptide epitopes in these studies suggest that the high concentrations of these cross-reactive antibodies are long-lived. This observation is also supported by flavivirus serodiagnostic studies identifying high levels of cross-reactivity in IgG assays [25], [28], [59]. Interestingly, although 4–7 months is not particularly long, the continued persistence of large populations of weakly or non-neutralizing cross-reactive antibody is relevant to antibody-dependent enhancement of infection and its potential role in increasing DENV disease severity leading to DHF/DSS [36], [60], [61]. Because antibody stimulated from EDII fusion peptide epitopes tends to be broadly cross-reactive, it is not surprising that the immunodominant nature of EDII fusion peptide epitopes is also supported by recent studies of WNV-infected human sera [26], [62].

We examined both primary and secondary DENV-2 infected patient sera and found EDII fusion peptide specific IgG was greater in primary than in secondary infections (mean = 44% and 35% respectively; Table 6). Lai et al recently used western blot to examine late acute-early convalescent phase DENV-2 sera from Taiwan [58]. They also found that the majority of the cross-reactive antibody response targeted epitopes in the highly conserved EDII fusion peptide. Moreover, Lai et al observed that the EDII-fusion peptide specific antibody response was greater in primary than in secondary DENV-2 infected patients, although they assayed total immunoglobulin and did not distinguish between IgM and IgG in their assays. The results presented in this report suggest that the increased cross-reactivity observed in primary relative to secondary DENV-2 infected sera results more from IgM than from IgG. IgM populations targeting these cross-reactive epitopes in primary and secondary infections were larger than IgG populations, and EDII fusion peptide specific IgM averaged twice as large in primary infected sera (54% and 24% of IgM in primary and secondary infections respectively, Table 6).

IgM responses to cross-reactive EDIII epitopes were similar in variability and magnitude as were those for the EDII fusion peptide, but the proportion of IgG recognizing these epitopes was much smaller (Table 5 and 6). Flavivirus immune responses targeting EDIII have not been examined in humans to the extent that they have for EDII fusion peptide epitopes, and neither of the DENV-2 infected patient sera studies cited above examined immune responses to EDIII. Oliphant et al recently studied epitope-specific immune responses in convalescent phase WNV-infected patient sera, examining both total EDIII IgG and EDIII WNV-specific IgG, the latter epitope being homologous to that examined for DENV-2 in this report [26]. Total IgG recognizing EDIII epitopes in the WNV sera averaged 7.3% and ranged from 0.6% to 50.5%, suggesting that the relatively small yet variable magnitude cross-reactive EDIII IgG response we estimated from DENV-2 infected patient sera (mean = 5.8%, range <1%–26%) could be a generalized response to flavivirus infection. More studies are needed to determine if the larger magnitude cross-reactive IgM response relative to IgG and the greater cross-reactive EDIII IgM in secondary relative to primary DENV-2 infections found in this study are typical for dengue or other flaviviruses. If so, the use of IgM titers or P/Ns alone to attempt to assign current infecting serotype in secondary DENV infections could be highly misleading. This observation is supported by detailed IgM and IgG analyses of a large number of primary and secondary DENV infected serum specimens (Chang et al, unpublished results).

Lai et al [58] examined late acute to early convalescent DENV-2 infected human serum specimens from Taiwan, similar to the Taiwanese serum specimens from this study. Using western blot to assay total immunoglobulin they estimated the DENV-2 specific anti E protein response in primary DENV-2 infections to range from 0% to 8.6% with a single specimen estimated at 21.5%. They did not attempt to identify the epitopes recognized by this DENV-2 specific immunoglobulin. Our results in primary DENV-2 infected patient sera were similar (<1%–9%) and indicate that the majority of this DENV-2 specific immunoglobulin is IgM (mean = 4% and <1% for IgM and IgG). We found the same pattern in secondary DENV-2 infected patients, but the magnitude of IgM and IgG responses averaged twice as large as in primary infections. The similar ranges of total DENV-2 specific immunoglobulin identified by Lai et al and of EDIII virus-specific IgM and IgG identified in the present study, suggest the possibility that DENV-2 specific epitopes in EDIII might constitute and elicit the majority of the virus-specific immune response in humans. Oliphant et al also found small proportions of EDIII recognizing virus specific IgG in WNV infected sera (mean = 1.6%, range 0%–6.8%), they did not however investigate WNV-specific epitopes outside of EDIII [26].

We were able to demonstrate for the first time that there is a significant and positive correlation between the magnitude of EDIII DENV-2 specific IgG titer and the percent of DENV-2 specific neutralization in humans. In WNV however, there was no correlation between overall levels of EDIII WNV-specific IgG and clinical outcome or measurable difference in neutralization profiles between WT and EDIII WNV-specific knock-out reporter virus particles using a flow-cytometric assay, suggesting a limited role for EDIII virus-specific IgG in WNV protective neutralization [26]. The apparent discrepancy between the importance of EDIII virus specific IgG as a correlate of protection in DENV-2 and WNV in these studies could result from the different neutralization assays that were utilized. One possible biological explanation for this discrepancy is the large titer differences between the two antibody populations in these studies, despite averaging about 1% of the IgG response against each virus. Total DENV-2 IgG titers, and hence EDIII DENV-2 specific IgG ranged from 10–100-fold greater than WNV IgG in the primary DENV-2 infected sera and up to 1000-fold greater in the secondary DENV-2 infected patient sera. A definitive test to see if these minor IgG populations are indeed protective would be to examine DENV-2 or WNV re-exposed vaccinee sera and determine if there is a marked anamnestic increase in this antibody population that is associated with increased protection. Nevertheless, our results suggest that the EDIII virus-specific epitopes stimulating strongly neutralizing and protective IgG in mice could play a similar role against DENV-2 infection in humans. The conclusions and questions stemming from the results presented in this report begin to disentangle the complex polyclonal humoral immune responses to primary and secondary DENV infections and point a direction for future studies in this field that will be essential both for improving our understanding of DENV pathogenesis and for the development of DENV vaccine candidates that may reduce the potential risk of antibody-dependent enhancement in vaccinees.

## Materials and Methods

### Cell culture, construction of plasmids and virus-like particle (VLP) production

COS-1 cells (ATCC CRL 1650; Manassas, VA) were grown at 37°C with 5% CO_2_ on Dulbeco's modified Eagle's minimal essential medium (D-MEM, GIBCO, Grand Island, NY) supplemented with 10% heat-inactivated fetal bovine serum (FBS, Hyclone Laboratories, Inc., Logan, UT), 110 mg/l sodium pyruvate, 0.1 mM nonessential amino acids, 2 mM L-glutamine, 20 ml/l 7.5% NaHCO_3_, 100 U/ml penicillin, and 100 ug/ml streptomycin. We used the recombinant expression plasmid pVAXD2i derived from pCBD2-2J-2-9-1 which been previously characterized and described in detail [20], [63]. This derivation is the same as same as described for constructing pVJE from pCBJE previously [25]. COS-1 cells were electroporated with WT pVAXD2i and mutant plasmids using the protocol described in [64]. Electroporated cells were recovered in 50 ml DMEM, seeded into three separate 75 cm^2^ culture flasks for VLP expression and incubated at 28°C with 5% CO_2_. Tissue-culture medium was harvested 4–5 days post transformation for VLP antigen characterization including triplicate measurements of secretion levels and MAb reactivity screening.

### Selection and introduction cross-reactive epitope residue substitutions

Cross-reactive epitope residues selected for substitution in the fusion peptide eliciting cross-reactive antibodies were originally identified by Crill and Chang, [20] with subsequent publications emphasizing the importance of overlapping immunodominant epitopes in this region [19], [26], [29], [43], [44], [45], [46]. Prospective EDIII cross-reactive epitope residues were also selected based on previously described procedural algorithms [20], [44]. Using this structure-based design approach we identified 22 different probably EDIII cross-reactive epitope residues. We modeled the effects of side-chain substitutions at eight of these residues using the DENV-2 Protein Data Bank E-protein structural coordinates (accession number 1OAN) and the swiss-model workspace selecting those with the highest probability of disrupting antibody binding without altering E-glycoprotein structural conformation, particle formation, or secretion [15] (http://us.expasy.org/spdbv/). Applying these criteria we selected 15 amino acid substitutions to introduce at these eight EDIII residue positions (Table 1).

Site-specific mutations were introduced into the DENV-2 E gene using the Stratagene Quick Change® multi site-directed mutagenesis kit (Stratagene, La Jolla, CA) and pVAXD2i as DNA template following the manufacturer's recommended protocols. The sequences of the mutagenic primers used for all constructs are listed in Table 1. Structural gene regions and regulatory elements of all plasmids were sequenced entirely upon identification of the correct mutation. Automated DNA sequencing was performed using a Beckman Coulter CEQ™ 8000 genetic analysis system (Beckman Coulter, Fullerton, CA) and analyzed using Beckman Coulter CEQ™ 8000 (Beckman Coulter) and Lasergene® software (DNASTAR, Madison, WI).

### Characterization of wild-type (WT) and mutant pVAXD2i secreted VLP antigen

Antigen-capture ELISA (Ag-ELISA) was used to detect and quantify secreted antigen from the mutagenized and WT pVAXD2i transformed COS-1 cells. Secreted antigen was captured in the inner 60 wells of Immulon II HB flat-bottom 96-well plates (Dynatech Industries, Inc., Chantilly, VA) with polyclonal rabbit anti-DENV-2 WT VLP hyper-immune sera diluted 1∶500, incubated overnight at 4°C, and wells were blocked with 300 µl of StartBlock blocking buffer (Pierce, Rockford, Ill.) according to the manufacturer's recommended protocol. Antigen was diluted 2-fold in PBS, incubated for 2 hr at 37°C and detected with murine hyper-immune ascitic fluid (MHIAF) specific for DENV-2 diluted 1∶2000 in 5% milk/PBS. Both the polyclonal capture and detector sera are hyper-immune fluids produced through repeated immunization and therefore containing high-titer immunoglobulin recognizing all potential antigenic epitopes. These polyclonal fluids are used at high concentration to insure that all VLP antigens, even those with dramatically altered epitopes, should be captured and detected similarly. MHIAF was detected using horseradish peroxidase conjugated goat anti-mouse IgG (Jackson ImmunoResearch, Westgrove, PA) in 5% milk/PBS and incubated for 1 hr at 37°C. Bound conjugate was detected with 3,3′5,5′-tetramethylbenzidine substrate (TMB; Neogen Corp., Lexington, KY), the reaction was stopped with 2N H_2_SO_4_ and measured at *A*_450_ using a Synergy HT Multi-Detection Microplate Reader (Bio-Tek Instruments, Inc., Winooski, VA). WT and mutant antigens were screened against the MAb panel using the same ELISA protocol as above with the exception that 2-fold dilutions of the specific MAb replaced the anti-DENV-2 MHIAF and antigens were used at a single standardized concentration producing an optical density (OD) of 1.0 in the secretion ELISA. Standardized concentrations of WT and mutant VLP antigens were analyzed in Ag-ELISA to determine MAb end point reactivities [18].

### Monoclonal antibodies

MAbs 4G2, 6B6C-1, 4A1B-9, 1B7, D3-5C9-1, 1A1D-9, 9D12, 10A4D-2, 1B4C-2, 9A3D-8, and 3H5 were obtained from the Arbovirus Diseases Branch, Division of Vector-borne Infectious Diseases, US Centers for Disease Control and Prevention (CDC). Many of these MAbs originated from the work of John Roehrig, 4G2, 1B7, 9D12, and 3H5 hybridomas were originally obtained by the CDC from the Walter Reed Army Institute [65]. MAbs 23-1, 23-2, 20, 5-1 and 5-2 were provided by Dr. L.-K. Chen, Tzu Chi University, Hualien, Taiwan.

### Human DENV-2 infected sera and basic serological characterization

DENV-2 infected human sera from dengue fever patients were obtained from the Dengue Branch, CDC, San Juan, Puerto Rico; and also from the Taiwan Center for Disease Control. The Puerto Rican DENV-infected patient sera were collected from Puerto Ricans infected locally during the 2007 transmission season and the Taiwanese sera were from Taiwanese residents who contracted DENV-2 while traveling in SE Asia outside of Taiwan in 2005. All sera were confirmed DENV-2 positive by virus isolation or RT-PCR either from these specimens or from paired acute-phase sera (data not presented and collected in either Puerto Rico or Taiwan).

Sera were assayed for the presence of E-specific immunoglobulins with both IgM and IgG antigen capture ELISA (MAC- and GAC-ELISA). All VLP antigen concentrations were standardized using the same antigen-capture described above in antigen characterization section. Antigen concentrations were standardized at an OD of 1.4, within the region of antigen excess near the upper asymptote of the sigmoidal antigen dilution curve (antigen is still the limiting factor here compared to the high concentration polyclonal rabbit serum and MHIAF used to capture and detect respectively). MAC- and GAC-ELISA were performed as previously described with some modifications [29]. Briefly, Immulon II HB flat-bottom 96-well plates were coated overnight at 4°C with goat anti-human IgM or IgG (Kirkegaard & Perry Laboratories, Gaithersburg, MD) and blocked with StartBlock. DENV-2 infected patient sera and positive and negative control sera were diluted 1∶1000 in wash buffer, added to wells and incubated at 37°C for 90 min. WT DENV-2 and negative control antigens were diluted appropriately in wash buffer tested against each serum sample in triplicate and incubated overnight at 4°C. DENV-2 virus-infected MHIAF was diluted 1∶2000 in 5% milk/PBS and incubated for 1 h at 37°C. Horseradish-peroxidase conjugated goat anti-mouse IgG was diluted in 5% milk/PBS, 50 µl, added to wells and incubated for 1 h at 37°C. Bound conjugate was detected with TMB substrate and plates incubated at room temperature for 8 min. The reaction was stopped with 2N H_2_SO_4_ and ODs were measured at *A*_450_.

P/N ratios were calculated as previously described with test validation utilizing internal positive and negative control sera on each plate [66]. Positive values were determined as the average OD for the patient serum sample reacted with WT DENV-2 antigen and negative values as the average OD of the normal human control serum with WT DENV-2 antigen. The primary or secondary DENV infection status of each specimen was determined by calculating the ratio of IgM/IgG OD each subtracted by two times the corresponding negative value; ratios ≥1.2 are indicative of primary infections and ratios <1.2 of secondary infections, Table 3 [67]).

### Determination of E protein epitope-specific immunoglobulin populations in DENV-2 infected human sera

To quantify the epitope-specific humoral immune response following DENV-2 infection we used the same MAC- and GAC-ELISA format described above and serially diluted sera from 1×10^3^ to 1×10^8^. In addition to WT DENV-2 VLP antigen we utilized a series of epitope-specific knock-out VLPs characterized in this study (Table 2). Relative concentrations of WT and epitope-specific knock-out antigens were standardized using the same capture ELISA format described in the P/N analysis above. Sera were diluted and tested with WT, five different epitope-specific knock-outs, and negative antigen in duplicate at each serum dilution. The five mutant DENV-2 VLP antigens used were an EDII fusion peptide G106R-L107D cross-reactive knock-out mutant (EDIII_FP_), an EDIII K310E-E311R-P364R complex cross-reactive knock-out mutant (EDIII_CR_), an EDII−EDIII combination of these two mutants G106R-L107D-K310E-E311R-P364R (EDII_FP_−EDIII_CR_) and two single EDIII mutants K305E and K388D (Table 2).

The resulting OD data were modeled as a nonlinear function of the log_10_ dilution using a four-parameter logistic model. In a four parameter logistic curve, the lower horizontal asymptote is not constrained to zero. This model was chosen over the three parameter model (which does constrain the lower asymptote to zero) because although the sera can be diluted to the point where there is no remaining immunoglobulin, the OD values are not expected to reach zero. The model was fit with the S-plus (v8.0) function nls (non-linear least squares) which uses least squares to estimate the parameters. Sera, imunoglobulin type, and antigen type were included as covariates. After fitting the model, endpoints were computed by finding where the OD curves for each antigen type crossed the OD curve for two times the negative control values at each serum dilution. The asymptotic covariance matrix ***V*** for the endpoints was computed using the multivariate delta method [68]. Using ***V***, general least squares was used to regress endpoints on antigen type, IgM/IgG, primary/secondary infection status, and Taiwan/Puerto Rico geographic origin class. Statistically significant main effects and two- and three-way interactions were investigated further with multiple comparisons using Scheffe's method [69].

The model could not satisfactorily fit the data for Taiwan sera #16 into a logistic curve, due to its very low positive OD signal, since our highest concentration serum dilution (1∶1000) only captured the tail end of the actual curve of signal vs. antibody concentration. Nevertheless, for both WT and all three EDIII mutant antigens there was positive OD signal greater than two times the negative value at the higher serum concentrations. For this serum sample we therefore used the following linear interpolation of the data to estimate the endpoint cut-offs, endpoint = (mean OD of the antigen of interest at the last positive serum dilution divided by two times the negative antigen OD at the same serum dilution) multiplied by the last positive dilution. Hence this is the percent remaining positive signal at the last positive dilution times that last positive dilution.

Epitope-specific IgM and IgG percentages were calculated by dividing the immunoglobulin end-point value obtained with a specific knock-out antigen by that obtained with the WT antigen on the same sera, subtracting this value from 1.0 and multiplying by 100. The two EDIII single substitution antigens were included to examine human immune responses to the protective EDIII virus-specific neutralizing epitope were analyzed slightly differently. K305E knocks out reactivity to neutralizing DENV-2 type-specific MAbs (e.g. 3H5 in this study) and to complex cross-reactive MAbs such as 1A1D-2; Table 2, [23], [47]. K388 is not incorporated into the DENV-2-specific epitope and knocks out only complex cross-reactivity exemplified by MAb 1A1D-2 (Table 2, Fig. 2; [47]. Thus, the percent immunoglobulin recognizing the DENV-2 specific EDIII neutralizing epitope was calculated as 100×1.0-[(K305 endpoint/WT endpoint)-(K388D endpoint/WT endpoint)]. In a few cases the titer measured with a mutant antigen was the same or greater than that with the WT antigen. We interpreted these cases as undetectable levels of antibody recognizing this epitope and the percent serum antibody recognizing this epitope was conservatively set to 1% for further analysis.

### Virus Neutralization Assays

We utilized an immunostaining focus-reduction micro-neutralization assay (FRμNT) to measure the neutralizing capability of the human serum samples against all four DENV serotypes, JEV and WNV. 2.47×10^4^ Vero cells in DMEM were added to 96 well black, clear flat bottom plates and incubated 16 hr overnight at 37°C and 5% CO_2_. Serum specimens were diluted 1∶50 in BA-1 and heat inactivated at 56°C for 30 minutes, diluted 2-fold to a final dilution of 1∶1600 and 320 virus pfu was added to each serum dilution. Plates were then incubated for 1 hr at 37°C, 5% CO_2_. After incubation, serum and virus suspensions were transferred back to Vero cell monolayer containing plates. These Vero cell plates were incubated at 37°C, 5% CO_2_ for 45 minutes rocking every 5 minutes to allow for virus infection. Barry's Ye Lah overlay media containing 6% sodium bicarbonate and 1% Carboxymethylcellulose sodium salt (Fluka biochemical) was added and plates were incubated at 37°C, 5% CO_2_. Incubation times were as follows: WNV (NY-99), JEV (SA-14-14-2): 24 hr; DENV-2 (16681) and DENV-4 (H241): 48 hr; DENV-1 (56BC94/95), DENV-3 (116RC1396): 70 hr. Following incubation plates were washed and fixed with 3∶1 acetone, then decanted and plates were allowed to dry overnight. Immunostaining was performed by adding virus-specific MHIAFs diluted in PBS and incubated at 37°C for 30 min, washing and adding goat anti-mouse HRP diluted in 5% milk/PBS and similarly incubated for 30 min. and washed. Infected virus foci were visualized using Vector-VIP peroxidase substrate kit SK-4600 as per manufacturers' instructions. Foci were counted using Zeiss KS300 microscope and Axiovision software version 4.6. 90% FRμNT dilution titers were calculated for each virus relative to that virus back-titration in BA-1. The actual 90% neutralization titers were calculated using Graph pad Prism version 4 (Graph Pad Software, San Diego, CA) sigmoidal dose response (variable slope) formula. All reported values are the average of two independent replicates.
